# Practitioners and Earning Power

**Published:** 1916-12-30

**Authors:** 


### PRACTITIONERS AND EARNING POWER.

No living man, being a worker, has in the whole course of his life ever experienced the burden of responsibility or the demand upon his faculties, to anything like the extent to which he has felt both during the closing months of 1916. Especially has this been the case where he has had the responsibility of keeping an organisation together and providing for the supply of brain workers and ideas.

The great underlying cause of the burdens in question lies in the effects of decades of years of continuous prosperity in this country, resulting in an absence of motive for continuous work on the part of a multitude of individuals, which has resulted in the loss to the nation of the products of valuable brains. Medicine has always been regarded as a learned profession, and practitioners as men who are bound by their oath to attain a high moral standard of excellence, and to keep always at their best. This tradition, though it has been a valuable asset to the profession of medicine, has deprived it of advantages open to workers in other professions. We have held, and from a life's experience still maintain, that there is no profession in the world which, properly understood, properly mastered, and properly utilised, can afford the individual worker the manifold openings and infinite variety of interesting fields of work which are available to a wise practitioner who has had the good fortune to learn something of business methods from his guardians or parents, and has kept them wisely in mind as a student and afterwards. Yet the profession, especially nowadays, does not provide all prizes for its members, however industrious they may be. It may be well to pause on the eve of 1917 to consider some underlying causes of failure, and to ask the attention of old and young practitioners alike to a consideration of the question of earning power in man.

Taking some causes of failure or ill-success first, every experienced practitioner will agree that one is the case of men who, on acquiring a qualification without having had the advantage of holding a house appointment, or some of them, in a busy hospital, proceed at once to commence private practice. Such men can have no adequate practical knowledge of diagnosis and treatment to warrant them in taking the risk of such a venture in such circumstances. Morally, no man is justified to enter upon practice thus ill-equipped and without the knowledge and experience which should be his before he invites patients to employ him and trust him, no matter what may be the nature of the illness or accident from which they may suffer. A young fellow who takes such risks deserves to fail, because a plea of necessity based upon business considerations cannot be justified in the business sense, and should properly be foredoomed to failure. It is, in fact, a risk which it is quite unnecessary for an intelligent man to suffer, because the opportunities which are open to every qualified man to get work which will keep him, whilst he is perfecting himself in the treatment of disease and the mastery of his profession, should be easily within his compass. Another danger which young practitioners are tempted to incur may be due to a realisation that to start as a surgeon, however ill-equipped, is to secure patients at once who will pay fees not otherwise obtainable. The growth in surgery, during the last decade especially, has caused older men who have not heretofore laid themselves out for surgery to undertake it, to practise and pursue it, and to defend this practice. All these things, in our judgment, are lamentable. They are matters which concern the well-being of the profession as well as that of the individual practitioner and his patients. They also have important public bearings. Hospitals with medical schools have continuously increased the number of their special departments, and we should like to see one more added to every such hospital—namely, that of Controller and Adviser of the Senior Students. The duty of the Controller should be to know each man's capacity and knowledge, and for the honour of the school and for the protection of the young practitioner to provide that the latter should be adequately equipped, in the professional sense, for practice before he enters upon it.

But what of the earning power in men? Has this question ever really engaged the continuous attention of either teachers or students? Once a qualification is obtained, how many men are there who have ever given a thought or ever had their thoughts directed to the study of earning power? If we could arrive at a system which would secure that the relative earning power of every qualified practitioner on entering the profession, and indeed of all practitioners, should be ascertained, we make bold to think, from a life's experience, that no failures to succeed, either material or professional, need arise. We have had exceptional opportunities to consider the earning power of individuals for decades of years. We have had evidence of the enormous loss of revenue which arises from a non-appreciation of this fact as it affects a man's success in life. We make bold to say that the first great medical school which appoints a Controller and Adviser for the purposes we have already indicated, provided infinite care is taken to secure a really competent holder of this new office, should within a decade have the largest entry of any medical school, and probably attain to the first position amongst practitioners' training-schools.

And what of the practitioners to-day who are willing to work and anxious to earn, but who find life a continual struggle and a heavy responsibility? This is too wide a matter to enter into fully in the present article. But we may say this, that to-day practitioners with a thorough knowledge of their profession or any branch of their profession, and especially those who have a wide knowledge of its many branches, can earn a very respectable living, if they have the gift of industry, can write the King's English, and possess a willingness to work and learn and earn. It may be an encouragement to our wide circle of readers to offer these considerations to them as our foreword for the New Year. Those who are interested, but have no immediate necessity, might call the attention of other members of the profession to this article. We are prepared to do our part to help anyone genuinely interested, if he or she is moved to make an appointment with the Editor, having first carefully thought out his position and difficulties, and then to come to him in good faith for such help as he is most willing and may be able to give.

